# Optical and Nonlinear Properties of Photonic Polymer Nanocomposites and Holographic Gratings Modified with Noble Metal Nanoparticles

**DOI:** 10.3390/polym12020480

**Published:** 2020-02-21

**Authors:** Oksana Sakhno, Pavel Yezhov, Volodymyr Hryn, Valentyn Rudenko, Tatiana Smirnova

**Affiliations:** 1 Fraunhofer Institute for Applied Polymer Research, Science Campus Golm, Geiselbergstr. 69, 14476 Potsdam, Germany; 2 Institute of Physics of NASU, pr. Nauky 46, 03028 Kiev, Ukraine; pviezhov@gmail.com (P.Y.); vohryn@iop.kiev.ua (V.H.); val@iop.kiev.ua (V.R.)

**Keywords:** photocurable nanocomposite materials, noble metal nanoparticles, photopolymers, holographic volume gratings, holographic recording, optical and nonlinear properties of nanocomposites and gratings

## Abstract

Nanocomposites based on transparent polymer matrices containing nanoparticles (NPs) of noble metals are modern-day materials that can be specially designed for photonics, linear and nonlinear optics, laser physics and sensing applications. We present the improved photosensitive nanocomposites doped with Au and Ag NPs allowing fabrication of high effective submicrometer dimensional diffraction structures using holographic method. A general approach for the fabrication of holographic structures using a two-component mixture of the monomers of different reactivity was developed. Two different methods, *ex situ* and *in situ*, were studied to introduce Au and Ag NPs in the polymer matrix. The diffusion model of the grating formation upon holographic exposure as well as the process of Ag NP synthesis in a polymer matrix is considered. The influence of the NP size on the polymerization process, material dynamic range and nonlinear properties were investigated. The mechanisms and characteristics of the nanocomposite nonlinear optical response are discussed.

## 1. Introduction

The nanocomposites based on optically transparent organic matrices with introduced noble metal nanoparticles (NPs) are one of the active areas of research in optics and optoelectronics. Their unique optical and electronic properties are mainly associated with the effects of dimensional quantization and dielectric restriction, as well as the excitation of local surface plasmons in metal NPs [1,2,3,4]. Nanocomposites based on doped glasses and polymers are used as materials for linear and nonlinear optics, laser physics and for sensing applications [1,2,3,4,5,6,7,8]. The polymer matrices have a number of advantages compared with glasses. They are relatively cheap, the methods of polymer synthesis are mostly well developed, and polymers provide high variability of their chemical structure and properties.

The linear and nonlinear optical properties of nanocomposites containing metal NPs are determined by the excitation of local surface plasmons in NPs, i.e., collective oscillations of the conduction electrons. The spectral position and intensity of plasmon resonance is very specific for different metals and depends strongly on both the properties and spatial organization of metallic NPs and the properties of the surrounding dielectric matrix [9,10,11,12,13,14]. Thus, by changing the shape, size and concentration of NPs, as well as the dielectric constant of the matrix, one can control the optical properties of nanocomposites. Polymer matrices are particularly well suited for implementation of such a control.

Plasmon properties of nanocomposites are also strongly influenced by a sub-micrometer scale ordering of the NPs [15,16]. In addition, periodic structures based on metal NPs possess great potential for applications in various fields of science and technology. Examples include the creation of optical memory and neural networks [17], a new type of diffraction elements with ultrahigh spectral dispersion [18,19] or distributed feedback lasers [20]. Another important application area of the metal-polymer ordered structures is the development of highly sensitive sensors for detecting small amounts of toxic substances [6,7,8,21,22]. In recent years, protection of the environment and human health from harmful pollutions has gained an even higher priority.

Nowadays, optical, nonlinear and plasmonic properties of NPs of various sizes and shapes and nanocomposites on their base are widely investigated, however this scientific direction is far from completion. Theoretical description of the systems of different sizes requires different approaches and does not always allow explaining and predicting the results of the experiment. New knowledge about nanosystems will enable to create improved theoretical models as well as to develop simple, effective and inexpensive technologies for the fabrication of materials and nanostructures with controlled properties that is a key-step towards their real applications.

In current work, we consider the photopolymerizable metal NP-polymer nanocomposites, which allow obtaining the volume structures with random or ordered NPs distribution using radical photopolymerization at a homogeneous or spatially modulated light illumination. Thus, a submicron periodic distribution of metal NPs within the matrix can be created using a simple one-step holographic method.

Periodic structures polymer-metal NPs can be fabricated with two methods. In the first case, the *ex situ* synthesized metal NPs are introduced into photocurable monomer mixture. Photopolymerization of the mixture with an interference pattern leads to spatial 3D ordering of the NPs in a polymer matrix. The volume diffraction structures are formed during light exposure and stabilized after a complete solidification of the layer. The second, *in situ*, method consists in introducing a metal precursor solution into the initial monomer mixture. In this case the NPs are formed after holographic recording from a laterally ordered precursor solution in the film with help of the subsequent UV exposure or heating. Both methods are shown by the example of new nanomaterials containing gold and silver NPs. The holographic and nonlinear optical properties of the presented nanocomposites and holographic structures will be reported. We note that in this paper we do not aim to fully reflect the studies of all known metal-polymer nanocomposites. The bibliography on this subject is huge and the basic principles and properties of the organic-inorganic and metal-organic nanocomposites are exhaustively shown in the review [23]. Here we refer only to the works directly related to the presented nanomaterials.

## 2. Experimental Methods

### 2.1. Sample Preparation and Holographic Transmission Setup

**Figure 1:**
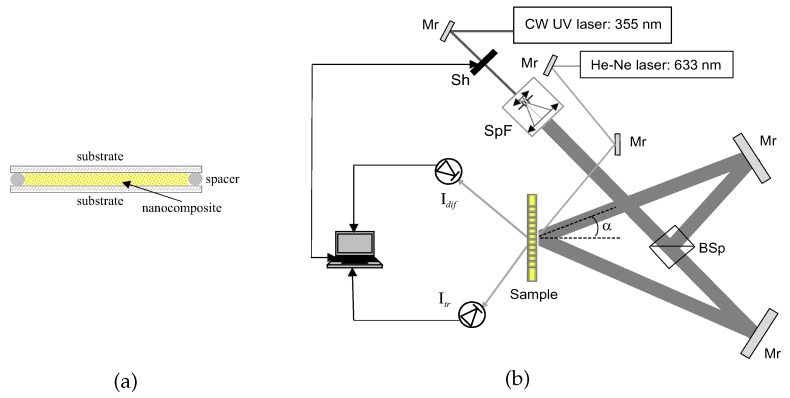
The sample preparation (**a**). Holographic setup for transmission gratings recording (**b**). Mr is the mirror; Sh is the shutter; SpF is the collimator with the spatial filter; BSp is the beam splitter; 2α is the angle between the recording laser beams; “sample” is the cell filled with the nanocomposite.

The photosensitive samples were prepared at room temperature, deposited a drop of the initially low-viscous nanocomposite between two glass slides of a proper size, using 20 µm thick Teflon spacers to provide the material layers of a uniform thickness (Figure 1a). The glass slides were previously cleaned in ethanol in an ultrasonic bath and were dried at 70 °C for of about 30 min in an oven. The samples were applied to holographic exposure almost immediately.

The experimental holographic setup is shown in Figure 1b. Two CW Ar^+^ lasers emitting at $\lambda_{r}$ = 364 and 488 nm (recording light) were used for holographic exposure. The *s*-polarised output beam was spatially filtered and collimated and after that split into two beams of the same intensity. The beam diameter was of about 15 mm. The two laser beams were spatially overlapped at the sample, forming the interference pattern inside the composite layer. The transmission gratings, prepared according to the *ex situ* method, are formed directly upon exposure to the interference pattern. Typical exposure time was between 80–200 s applying total intensity of two beams from 3 to 10 mW/cm^2^ dependently on the composite and its spectral properties. The period of the grating (Λ) was varied in the range of 0.5–1 µm by changing the angle between the recording beams (2α), $\Lambda =\lambda_{r}/2sin\alpha$ (Figure 1b).

A real-time monitoring of the grating formation process was provided with an unexpanded He-Ne laser beam (633 nm). The beam falls on the sample at the angle, corresponding to the Bragg angle of a current spatial grating period. During holographic exposure the signals of the diffracted −1^st^ order, $I_{dif}$, and the transmitted, 0^th^ order, $I_{tr}$, are pick-up with a data collection system. The current diffraction efficiency of the grating (η) is estimated from the experimental data as η(t) = $I_{dif}\left(t\right)/\left(I_{tr}\left(t\right)+I_{dif}\left(t\right)\right)$. The diffraction efficiency values are used for calculation the refractive index modulation amplitude, $n_{1}$, of the gratings.

The Bragg diffraction conditions are satisfied for the gratings with the specified parameters. Only the 0^th^ and −1^st^ diffraction orders were observed in the diffraction pattern. Therefore we used Kogelnik’s formula [24] for calculation of the amplitude of the refractive index modulation, $n_{1}$:

(1) $$\begin{array}{c} n_{1}\;=\;\frac{\lambda_{t}\;cos\theta_{B}\;asin\;\eta^{1/2}}{\pi \;d}, \end{array}$$

where $\theta_{B}$ is the Bragg angle within the material, $\lambda_{t}$ is the wavelength of the reading beam, *d* is the grating thickness.

The measurements were carried out for a series of five samples for each Au NP concentration. Typical deviations in the layer thicknesses in our experiments was Δd = ±1.2 µm and the deviation of the diffraction efficiencies was Δη = ±2%. Based on these results, the error of the obtained values of $n_{1}$ was calculated and shown in corresponding figures as the error bars.

In previous works, we have studied in detail the influence of various factors on the efficiency of holographic recording in the organic-inorganic photocurable nanocomposites [25]. It has been shown that the values of η and, finally, $n_{1}$ are determined not only by the material formulation but by the recording conditions also, namely, period and intensity (*I*) of the recording beams. During investigation of the grating kinetics only optimized exposure parameters were applied in order to ensure a maximum η of the gratings.

### 2.2. Measurement of Nonlinear Optical Performances

To study the nonlinear properties of the nanocomposites modified by Au and Ag NPs, the nonlinear refractometry and Z-scan methods, known in nonlinear optics, were used. The measurements were performed at the second harmonic wavelength of the Nd:YAG pulse laser with the following output characteristics: Pulse duration 20 ns, wavelength 532 nm, repetition rate 0.5 Hz.

The nonlinear refractometry technique is based on the recording of dynamic holographic gratings in a nonlinear medium. In this case two laser beams fall on the sample at a certain angle. During the action of the laser pulse, these beams interfere, forming a dynamic grating. Measuring the dependence of laser pulse energy diffracted at the first order on the energy of the pulse incident on the grating, the parameters of the medium nonlinearity can be calculated.

**Figure 2:**
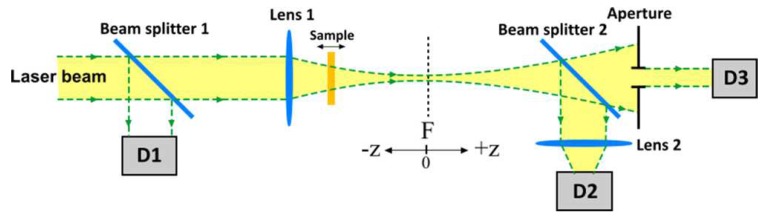
Scheme of the Z-scan setup, D1, D2, D3 are the photodetectors.

The principal scheme of the Z-scan is shown in Figure 2. The parameters of the setup: The lens focus is 10 cm; the aperture diameter is 0.5 mm; diaphragm transmission is 0.14; the beam waist radius is 68 µm. The dependence of the sample transmission on its position with respect to the beam waist (F) at a fully open and a limited aperture is measured. Processing of the results allows determining the nonlinear characteristics of the medium.

### 2.3. Other Measurement Methods

The polymerization kinetics of the monomer mixture and the nanocomposites was investigated with Fourier transform infrared spectroscopy (FTIR) using a Mattson Instruments RS 10000 FTIR. The initial mixtures were sandwiched between two CaF_2_ slides with a cell thickness of 250 µm. The light-curing was carried out with UV light from a Philips PL 10W-10 (with intensity of about 2 mW/cm^2^ and average wavelength of 365 nm).

A Perkin–Elmer Lambda 2 spectrometer was used to measure optical absorption spectra of the nanocomposite films. The thickness of the gratings and polymer films was measured by Dektack 100 profiler after removing one of the substrates. The refractive indices of the monomers and the nanocomposites were measured with help of Abbe refractometer.

## 3. Results and Discussion

### 3.1. Nanocomposites with Au NPs

#### 3.1.1. Development of the Nanocomposites with Au NPs and Their Properties

Tomlinson firstly proposed a general approach for the creation of photopolymerizable composites for holographic recording of phase volume periodic structures [26]. In order to ensure high amplitude of the refractive index modulation, the composite must include at least two components: High-reactive and low-reactive monomers, which polymerize independently at significantly different rates. The mechanism of the periodic structure formation is based on the diffusive redistribution of the components during the polymerization process under a spatially inhomogeneous light illumination. This causes a spatial modulation of the material formulation and, respectively, the refractive index of the medium. The low-reactive monomer can be also replaced by a chemically neutral component that does not interact with the components of the mixture, but participates in the diffusion mass-transfer. Later in the works of Tomita [23] it was shown that the NPs of different nature can act as a neutral component and can also be involved in the diffusion process. Despite the clarity of the basic principles, the creation of new nanocomposites always requires a choice of the composite components and optimization of the formulation.

Firstly Bunning et al. applied the Au NPs in the holographic photopolymers [27]. The reported holographic gratings of 20 µm thickness, containing the Au NPs concentration of about 5 wt. %, performed a weak, less than 0.01, diffraction efficiency, and, correspondently, low amplitude of refractive index modulation.

In the [28] we proposed own holographic Au NP-containing composite that allows increasing $n_{1}$. In the present work we improve the composite formulation and investigate the influence of NP size and plasmonic properties on the holographic and nonlinear optical characteristics. The choice of the photocurable components for holographic polymers is a crucial point. They must be a good dispersive media for the NPs, namely, must allow easy dispersion of the NP within the medium upon holographic patterning without forming the aggregates and ensure high stability of the final structure.

We relied on experience of the nanocomposites containing NPs of different nature (metal oxides, semiconductors, etc.) [29,30,31], when choosing the organic components for the formulation with the Au NPs. It was found that the best results were obtained by using the two-component mixtures of mono- and multifunctional acrylate monomers, which polymerize independently with substantially different rates.

**Figure 3:**
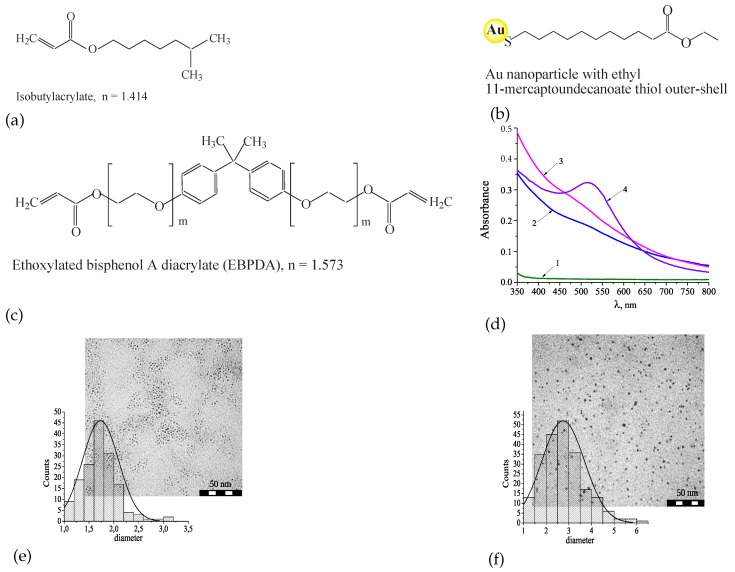
The structures of the monomers used in the nanocomposites and their refractive indices (**a**–**c**). Optical spectra of the layers (**d**): Without Au nanoparticles (NPs) (curve 1), and with different concentrations of Au NPs : 1.5 wt. % Au1 (curve 2); 2 wt. % Au1 (curve 3); 1.3 wt. % Au2 (curve 4); TEM image and histogram of the size distribution of Au1 NPs (area is 0.17 µm × 0.17 µm; average diameter is 1.7 nm; standard deviation is 0.36 nm) (**e**) and Au2 NPs (area is 0.19 µm × 0.17 µm; average diameter is 2.7 nm; standard deviation is 0.94 nm) (**f**).

The holographic syrups we employed in this investigation are homogenous mixtures of acrylate monomers and specially prepared ready-to-use Au NPs, i.e., *ex situ* method. The chemical formulae of the monomers used, their refractive indices, *n*, are shown in Figure 3a,c.

Isooctylacrylate (IOA) a low viscous, non-polar, low-shrinkage monofunctional monomer and ethoxylated bisphenol A diacrylate (EBPDA) a bifunctional low-shrinkage monomer generating insoluble cross-linked network were used in weight ratio IOA:EBPDA = 80:20. Free-radical photoinitiator Irgacure 1700 was added to the syrup to provide the sensitivity to UV light (365 nm). The Au NPs were firstly dispersed in CH_2_Cl_2_ and then mixed with the pre-polymer syrup. After evaporation of the solvent the final composites with Au NPs content (0–2 wt. %) were ready for holographic exposure.

To study the influence of the particle size on the holographic and non-linear properties of the composites, the NPs of two core sizes (Au1 and Au2) were prepared. The Au NPs were mostly spherical; the average NP core diameter was evaluated from TEM images. The Au1 and Au2 possess the core diameter of 1.7 ± 0.36 nm and 2.7 ± 0.94 nm, respectively (Figure 3e,f). The synthesis of the Au NPs and method of TEM measurements were described in detail in [28]. The surface of the Au NPs was functionalized with ethyl 11–mercaptoundecanoate (Figure 3b) that provides their high stability in solvent and in the holographic mixture while the ester functionality ensures good solubility.

Optical spectra of the layers without and with the Au NPs are shown in Figure 3d. The sample without Au NPs is almost transparent in the spectral range of 400–800 nm. The addition of the NPs (Au1) to the pre-polymer mixture leads to the increased absorption in the UV range and the formation of a weak shoulder at 520 nm. With an increase in the size of the NPs (Au2) a characteristic band with a maximum at near 520 nm appears which is associated with localized surface plasmon excitation in the Au NPs.

#### 3.1.2. Formation of Volume Periodic Structures Using the Au NPs Containing Composites

**Figure 4:**
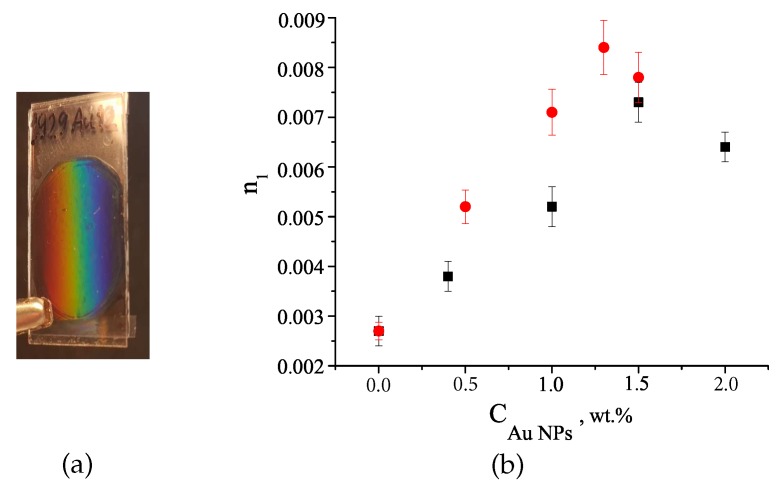
(**a**) Photo of the grating in the nanocomposite with Au NPs. (**b**) Maximum values of $n_{1}$ as a function of the Au NPs concentration for Au1 (black squares) and Au2 (red circles).

The dependence of the grating kinetics on the NP concentration (C_*NP*_) was studied for both, Au1 and Au2, NPs. The values of $n_{1}$ as a function of the Au NPs concentration is presented in Figure 4. The general peculiarities of the grating formation in the nanocomposites with Au NPs of different sizes remain unchanged. In the pre-polymer mixture without the NPs the formation of stable gratings with a maximum η of only 0.08 ($n_{1}\approx$ 0.0027) was observed.

The introduction of 1 wt. % of Au NPs to the pre-polymer mixture causes a significant growth of the grating η to approximately 0.23, in the case of Au1, and to 0.53, in the case of Au2 NPs. In both cases, an increase in the C_*NP*_ leads to an increase in $n_{1}$, which reaches its maximum value at an optimal concentration and then decreases with further increasing NP concentration. The grating based on the material with $C_{NP}$≈ 1.5 wt. % of Au1 shown the maximum value of $n_{1}$ of about 0.0073 (η = 0.55). The increase in the NP size leads to an increase in $n_{1}$ close to 0.0084 (η = 0.67) and to a decrease of the optimal $C_{NP}$ to of about 1.3 wt. %.

**Figure 5:**
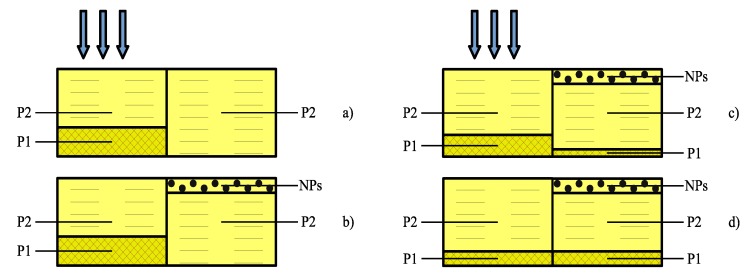
The model under consideration: (**a**) A two-component mixture, without Au NPs; (**b**–**d**) a three-component mixture containing Au NPs.

The obtained results make it possible to establish the contribution of the diffusion transport of each component to the formation of the periodic structure. We used a phenomenological model for the analysis [25,32]. In the initial state, the composite is an equilibrium mixture of the three components. The polymerization in the maximum of the interference pattern disturbs the equilibrium of the system, creating the gradient of the chemical potential for each component that, in turn, causes the diffusion redistribution of them between the illuminated and dark areas of the layer. As a result, these areas are differed by their chemical contents and average refractive indices, forming the phase image of the interference pattern. The model assumes that the redistribution of the components occurs at a constant volume of the system; the shrinkage of the polymer is not taken into account. We suppose that both monomers and the NPs participate in the diffusion mass-transfer. Figure 5 illustrates the model under consideration.

In the begining we will consider a two-component mixture comprising monomer M1 (EBPDA) and monomer M2 (IOA). The component redistribution, which provides the highest possible modulation of the refractive index is shown in Figure 5a. In this case, the polymer network P1, originated from M1, will be formed in the high-intensity areas. The areas corresponding to the minima of the interference pattern will be filled by the polymer P2, being formed from M2. The amplitude of the refractive index modulation of a two-component mixture can be approximately described by:

(2) $$\begin{array}{c} n_{1}\;=\;\frac{1}{2}\left|\left(n_{P1}-n_{P2}\right)\Delta \upsilon_{P2}\right|, \end{array}$$

where $n_{P1},n_{P2}$ are the refractive indices of polymers, formed from the first and the second monomer, $\Delta \upsilon_{P2}$ is the difference between the volume fractions of polymer P2 in both areas. At the mention weight fraction of the components, their volume fractions are equal 0.84 for IOA and 0.16 for EBPDA. The following refractive indices of the polymers were used for the calculations: $n_{P1}$ = 1.573 and $n_{P2}$ = 1.475. In the case presented in Figure 5a $\Delta \upsilon_{P2}$ = 0.32 and $n_{1}≅$ 0.0157. The relative modulation of the component concentration, $\Delta \upsilon_{i}^{*}/\upsilon_{i}$, where $\Delta \upsilon_{i}^{*}$ is the amplitude of the volume content modulation of the i-th component, is 100% for EBPDA and of about 19% for IOA. The obtained $n_{1}$ and $\Delta \upsilon_{P1}^{*}/\upsilon_{P1}$ are collected in Table 1. The maximum $n_{1}$ obtained from the experimental data and also presented in Table 1 is equal to 0.0027. Using Equation (2) the experimentally obtained value of $\Delta \upsilon_{P1}^{*}/\upsilon_{P1}$ was estimated as 17%. The found data suggest that in such a mixture there is no complete transfer of M1 towards the illuminated areas.

In the case of a three-component mixture including monomers and nanoparticles $n_{1}$ is determined as:

(3) $$\begin{array}{c} n_{1}\;=\;\frac{1}{2}\left[\right|\left(n_{P2}-n_{P1}\right)\Delta \upsilon_{P2}\left|+\right|\left(n_{NP}-n_{P1}\right)\Delta \upsilon_{NP}\left|\right], \end{array}$$

where $n_{NP}$ is the refractive index of NPs, $\Delta \upsilon_{NP}$ is the modulation of their volume content.

We have considered three cases: (i) Polymer of EBPDA is fully localized in the illuminated areas of the recording layer, the Au NPs are localized in the dark areas (Figure 5b); (ii) the modulation of EBPDA concentration is the same as in a composite without NPs, the Au NPs are localized in the dark areas (Figure 5c); (iii) only the Au NPs participate in the diffusion mass-transfer and they are localized in the dark areas (Figure 5d). The case when the Au NP move to the illuminated areas was not examined because in this case $n_{1}$ can only decrease.

In the composite containing 1.5 wt. % of Au1 NP, the mass-fraction of the monomers are equal to 78.8 and 19.7 wt. % for IOA and EBPDA, correspondently. The refractive index and density of the NP material were used to estimate these values. The real part of Au refractive index in the spectral range of 500–650 nm is $n_{NP}$ = 0.2 and density ρ = 20 g/mL [33]. Using Equation (3) we calculated $n_{1}$ and $\Delta \upsilon_{P1}^{*}/\upsilon_{P1}$ for all models (Table 1). The measured $n_{1}$ and corresponding $\Delta \upsilon_{P1}^{*}/\upsilon_{P1}$ values are also collected in the Table 1.

In the case shown in Figure 5b $n_{1}≅$ 0.0167, $\Delta \upsilon_{NP}$ = 0.00148, and the relative concentration modulation of the Au NPs and P1 is equal to 100%. The $n_{1}$ decreases to 0.0037 if the $\Delta \upsilon_{P1}^{*}/\upsilon_{P1}$ decreases to 17%, as it was experimentally measured for the two-component composite without Au NPs (Figure 5a). In the latter case, when the grating formation is determined by the Au NPs migration only $\Delta \upsilon_{P1}\;=\;0,\Delta \upsilon_{P2}\to 0$, $\Delta \upsilon_{NP}$ = 0.00148 and $n_{1}$ is equal of about 0.001. That is almost of one order of magnitude less than the measured $n_{1}$ values.

Finally, based on the obtained results the following conclusions can be done. If the Au NP content in the composite <2 wt. %, the diffusion redistribution and a complete localization of the Au NPs in the dark areas does not provide effective holographic recording. The main contribution to the formation of the periodic structure is reached due to increasing segregation of the polymer phases. Complete segregation of M1 and localization of P1 in the illuminated areas (relative modulation of P1 concentration is 100%) is not achieved, however, the monomer segregation increases when the Au NPs are introduced into the mixture. In the nanocomposite with 1.5 wt. % Au1 NPs $n_{1}$ = 0.0073 and $\Delta \upsilon_{P1}^{*}/\upsilon_{P1}\approx$ 38%, correspondingly. Increase of the particle size reduces the optimal concentration of Au2 NPs down to 1.3 wt. %. In this case, $n_{1}$ increases to 0.0084 and $\Delta \upsilon_{P1}^{*}/\upsilon_{P1}$, respectively, to about 42%. Thus, the relative modulation of P1 concentration in the composite with Au NPs is more than 2 times higher than that achieved by holographic polymerization of the mixture without the NPs. The influence of the Au NPs on the polymerization process will be discussed below.

#### 3.1.3. Influence of the Au NPs on the Photopolymerization Process

The impact of the Au NPs on photopolymerization of the monomer mixture was investigated using the photocuring kinetics of the composite without and with the Au NPs applying a homogeneous UV illumination. The degree of conversion of the material was evaluated with FTIR spectroscopy.

**Figure 6:**
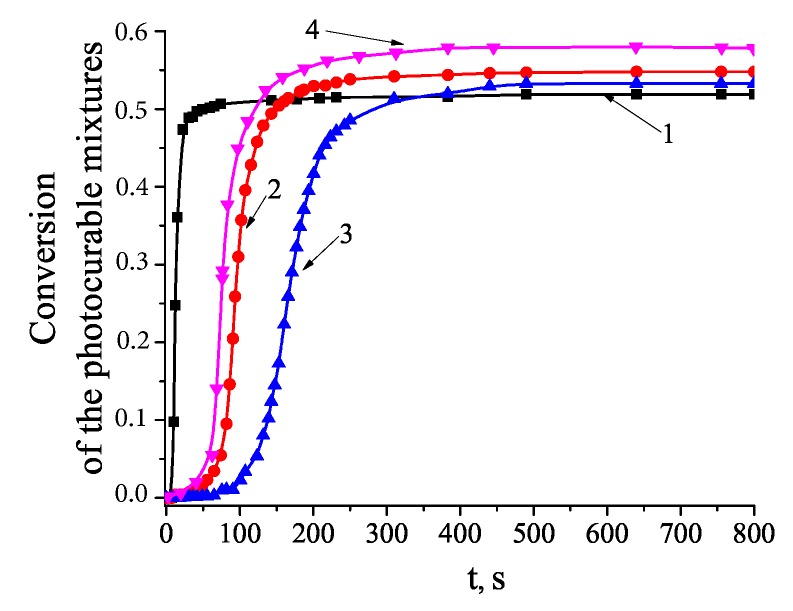
Conversion of: The monomer mixture (1); the nanocomposite with 1.5 wt. % Au1 (2) and 2 wt. % Au1 (3); the nanocomposite with 1.3 wt. % Au2 (4).

By monitoring with a short time-interval exposure the disappearance of the absorption band near 1640 cm^−1^ including vibrations of the aliphatic and aromatic C=C bonds and using the formula: $C\left(t\right)\;=\;\left(A_{0}-A\left(t\right)\right)/A_{0}$, (where $A_{0}$ and A(t)—absorption of the film before and during the UV-exposure, correspondingly) the degree of the material conversion was determined (Figure 6).

The kinetic dependencies for the monomer mixture and the compositions containing optimal concentration of the Au NPs of different size, 1.5 wt. % of Au1 and 1.3 wt. % of Au2, have been measured. The rate of polymerization is maximal for a pure monomer mixture and it decreases in the case of the monomer mixture containing the Au NPs with increasing concentration of the NPs. The depth of conversion of the polymer in the composite containing the Au NPs is higher than that of the mixture without the NPs. Moreover, the rate of polymerization and conversion depth increase when using the Au2 NPs of larger size.

From the theory of periodic structure formation in photopolymerizable materials [32,34] it is known that the maximum value of $n_{1}$ is achieved when the characteristic polymerization time, $\tau_{pol}$, exceeds the characteristic diffusing time, $\tau_{dif}$ (time of material transfer at a distance equal to the grating period): ($\tau_{pol}/\tau_{dif}$ > 1).

Low segregation of the components in the monomer mixture under study indicates that the polymerization rate of the monomer mixture does not meet this inequality (Figure 6, curve 1). The decrease in the polymerization rate of the mixture with the Au NPs promotes the component transfer and, accordingly, increases the spatial modulation of the component concentration. On the other hand, the change of polymer conversion depth with the addition of Au NPs confirms the specific influence of Au NPs on the formation of polymer network. There are many mechanisms, which allow changing the structure of the polymer network. In particular, catalysis of the radical reactions in the presence of colloidal metals is well known. Since polymerization of the acrylate monomers occurs through the free radical mechanism, the introduction of the Au NPs can influence on the rate of the elementary reactions of polymerization and, consequently, the polymerization kinetics and the density of the polymer network. It is noteworthy that despite the small difference in the size of the Au NPs (1.7 and 2.7 nm), the larger Au2 NPs significantly affect the kinetics of polymerization (Figure 6, curve 4). Au1 and Au2 have the same shape and the same outer-shell. Still these NPs are significantly different due to the excitation of localized surface plasmons in the Au NPs of larger size, while plasmon resonance is absent in the Au NPs of smaller size, Au1. Since the collective excitation of electrons in the NPs results in strengthening of a local field near the NP surface, it can be assumed that this can also affect the kinetics of photochemical reactions. It should be note that the Au1 NPs are much closer to the molecular clusters, which do not possess typical metallic properties; however, the general regularities of photopolymerization and the holographic recording process are the same for both Au1 and Au2 NPs. Generally, the effect of Au NPs of different size on the polymerization process and the molecular structures of the emerging polymers requires special studies.

### 3.2. Nanocomposite with Ag NPs

#### 3.2.1. Choice of the Composition Components and the Nanocomposite Fabrication

The *ex situ* method for the creation of periodic structures with noble metals NPs has a number of disadvantages. First of all, metal NPs aggregate rapidly in low-viscous monomers. To obtain a homogeneous mixture the NP surface must be decorated with a proper outer-shell, which prevent their aggregation. Such additional surface passivation complicates the manufacturing of the structures. Additionally, in order to record the structures with submicron periods, it is necessary to use laser sources with the emission wavelengths of 350–500 nm, which coincide with the plasmon absorption bands of the most commonly used metal NPs (Ag, Au, Cu).

This leads to absorption of the recording radiation and to inhomogeneity of the structure over the layer depth, thereby limiting an allowable concentration of the NPs in the initial monomer mixture.

These disadvantages can be overcome by using an *in situ* NP formation method, when a metal precursor is introduced into the monomer mixture. In this case the metal NPs are formed after holographic structuring of the composite. This also stabilizes the resulting structure. Since gold or silver precursor solutions absorb in the spectral region <350 nm, holographic exposure can be performed over the entire visible range. Besides that higher concentration of the forming NPs can be achieved than when the ready NPs are introduced to the monomer mixture.

In [35] we presented an *in situ* method for ordering of Ag NPs in polymer matrix and developed a suitable monomer mixture for this aim. In this work, we describe new photocurable monomer blend, which provides a significant increase in the refractive index modulation of the periodic structures polymer–Ag NPs. Below we analyse the grating recording mechanism, investigate the holographic characteristics of the proposed composite and optical nonlinear properties of the Ag NP doped polymer films.

The choice of the composition components for the *in situ* NPs formation of periodic distribution of Ag NPs in polymer is based on our previous studies of photopolymerizable composites including a neutral component (NC) [36].

**Figure 7:**
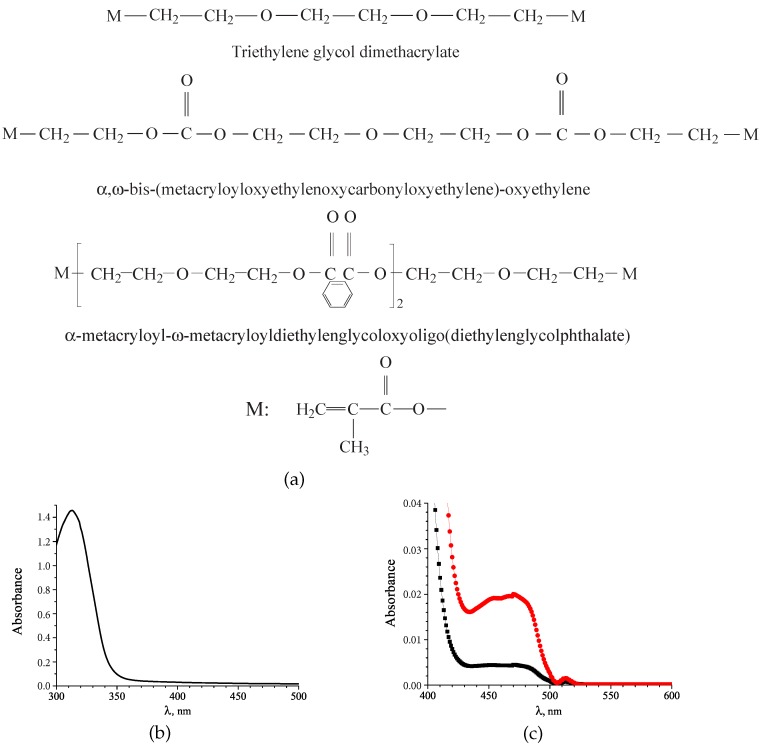
Chemical structure of the mixture components (**a**). Absorption spectra of a solution of AgNO_3_ in acetonitrile (**b**), and mixture before (red) and after (black) polymerization (**c**).

Based on these knowledge a mixture of bifunctional monomers triethylene glycol dimethacrylate (10 wt. %), α,ω-bis-(metacryloyloxyethylenoxycarbonyloxyethylene)-oxyethylene (44 wt. %), and α-metacryloyloxy-ω-metacryloyloligo(oxyethylene) (44 wt. %) were used as a photopolymerizable matrix. Solution of AgNO_3_ in acetonitrile (30 vol.% with respect to the monomers) was used as the NC. The photoinitiating system Michler’s ketone (MK, 1 wt. %) and camphorquinone (CQ, 5 wt. %) provides the sensitivity of the composite in the 440–500 nm spectral range. The structures of the composite components are shown in Figure 7a.

The photosensitivity of the composition is provided by the absorption of CQ. A low-intense absorption band at $\lambda_{max}$ = 450 nm corresponds to n→π energy transfer in the molecule of CQ (Figure 7c). A weak absorbance at $\lambda_{rec}$ = 488 nm (absorption coefficient k ≤ 5 cm^−1^ at optimal CQ concentration) provides the homogeneity of the holograms with the thickness of d ≤ 100 µm over the depth of the film. During polymerization the CQ peak is almost completely degraded.

#### 3.2.2. Recording Mechanism and Properties of Periodic Structures

Since the monomers included in the mixture are copolymerized during holographic exposure, the composite under study can be considered as a two-component one. It is established that during the holographic exposure the basic components, such as a monomer mixture and a solution of metal precursor, are involved in an irreversible photoinduced mass-transfer between the illuminated and dark areas of the layer thus ensuring the stability of the structure. The polymer network is formed and placed mainly in the grating fringes corresponding to the high intensity areas of the interference pattern. The precursor solution forces out of the polymer network and localized mostly in the low illuminated areas. Complete polymerization of the film provides the formation of a volume structure consisting of the periodically arranged regions: Polymer and polymer, enriched with a metal precursor. A subsequent heat treatment of the sample causes evaporation of the solvent and the formation of Ag NPs mainly in the areas containing a metal precursor.

**Figure 8:**
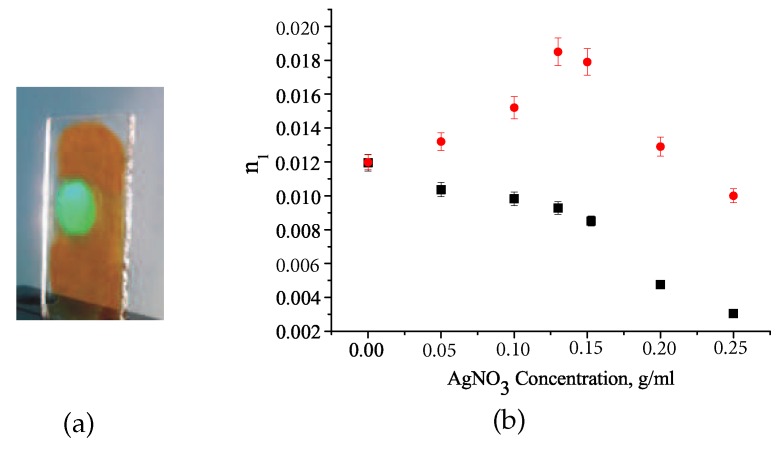
Photo of the grating polymer–Ag NPs (**a**). The dependence of $n_{1}$ of the grating before (black squares) and after (red circles) the treatment on the AgNO_3_ concentration in a precursor solution (**b**).

The composite formulation was optimized measuring the recording kinetics of the gratings prepared from the composites containing a precursor solution of different concentrations of AgNO_3_ (Figure 7). The $n_{1}$ of the grating polymer-Ag precursor continuously decreases with increasing concentration of AgNO_3_ (Figure 8). At the same time, $n_{1}$ of the grating containing Ag NPs increases, reaching its maximum value at the optimum concentration of AgNO_3_ and, finally, also decreases.

Using Equation (2), the influence of the metal precursor on the phase separation of the composite components and, accordingly, the efficiency of the polymer-precursor grating formation can be estimated. In this case, the first term in the right part of Equation (2) is the difference between the refractive indices of the polymer and NC ($n_{NC}$). The second term describes the difference between the volume concentrations of NC in the illuminated and dark areas of the layer, $\Delta \upsilon_{NC}$. The refractive indices of the polymer and pure acetonitrile are $n_{P}$ = 1.516 and $n_{NC}$ = 1.345, correspondingly. The value $n_{1}$ of the grating prepared from the monomer mixture with pure acetonitrile without AgNO_3_ is 0.012. It means that the relative modulation of the NC concentration is approximately equal to 47%. As the AgNO_3_ concentration increases, the precursor refractive index increases to 1.358 at optimal AgNO_3_ concentration of 0.13 g/mL. The relative modulation of the NC concentration reduces to approximately 39%. For the calculations, the experimental value of $n_{1}$ = 0.0093 was used. The observed decrease in the value of $\Delta \upsilon_{NC}^{*}/\upsilon_{NC}$ indicates a decrease in the NC displacement from the polymer network, which leads to a decrease in $n_{1}$. The reason that can explain the decrease in $n_{1}$ with increasing concentration of AgNO_3_ is the increase of thermodynamic compatibility of NC and the polymer matrix and, accordingly, the equilibrium concentration of NC in the polymer due to dissolution of AgNO_3_ in acetonitrile. A metal precursor can affect the polymerization process and properties of polymer network, as was observed in the case of materials with Au NPs. The latter requires further research. An increase in the concentration of AgNO_3_ is accompanied by a decrease in $n_{1}$, as well as an increase in light scattering in the grating. Therefore, the gratings were made from a composite containing the optimal concentration of AgNO_3_ (0.13 g/mL). This ensured the achievement of the maximum value of the $n_{1}$ at minimal light scattering in the gratings.

As mentioned above, the value of $n_{1}$ depends only on the thermodynamic properties of the polymer-NC system when $\tau_{pol}/\tau_{dif}>$ 1. The recording conditions (Λ<1 µm, *I* = 0.5 mW/cm^2^) were chosen so that the specified inequality holds.

The Ag NPs are formed mainly during thermal (UV) post-processing of the silver precursor/polymer gratings. After recording, one of the substrates, pre-treated with an anti-adhesive coating, was removed and the grating was kept at a temperature of 70 °C until a stable value of η was reached. Continuous monitoring of η made it possible to trace the grating changes. For a polymer-precursor grating with Λ = 0.9 µm, the initial value of $n_{1}$ was of 0.012. After removal of the substrate and evaporation of acetonitrile $n_{1}$ decreases to 0.0015. Further heating led to an increase of $n_{1}$ to its steady-state value of 0.021 that can be associated with the formation of Ag NPs.

**Figure 9:**
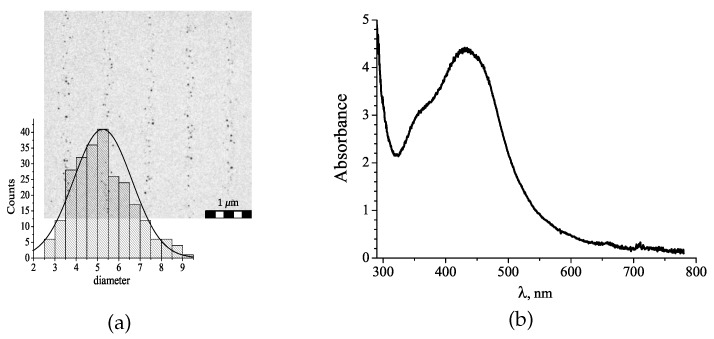
(**a**) TEM image of the grating with the *in situ* formed Ag NPs and histogram of the NP size distribution (the area is 3.5 µm × 3.5 µm; the average diameter is 5.25 nm; the standard deviation is 1.3 nm); (**b**) optical spectrum of the nanocomposite film containing Ag NPs.

The formation of the NPs was confirmed by transmission electron microscopy (TEM) measurements and by the changes in optical spectrum the gratings containing the Ag NPs. The TEM measurements show a periodic distribution of the NPs in the polymer matrix. The experimental technique was described in [35] in detail. In a micrograph (Figure 9a) one can see that spherical NPs with an average diameter of about 5.25 nm form the periodic grating fringes. The size of the NPs varies in a range of 2.5–9 nm. The NPs with a size <2 nm were not detectable.

We would like to note that a 100% modulation of the Ag-precursor within the grating is not achieved; therefore, we expected a more uniform NP distribution within the matrix. However, we do not observe any NPs in the spaces between the areas of their main location. It is known that processes of Ag NPs photoreduction and grows are diffusion controlled. Thus, a high-density polymer network forming in the high intensity regions of the interference pattern can prevent the formation of the NPs with diameter >2 nm.

The absorption spectrum of the holographic structure changes significantly after heat treatment. The polymer film containing metal precursor and the photoinitiator remains almost completely transparent in the 400–500 nm spectral range (Figure 7c). After heat-treatment the structure reveals significant absorption in the same range. Two spectral bands are observed in the corresponding spectrum. The band with a maximum at 360 nm belongs to the MK photoinitiator. The spectral band with a maximum near 450 nm is a characteristic feature of a localized surface plasmon excitation in AgNPs, that confirms the formation of Ag NPs in the grating structure.

The replacement of the composite photoinitiator system opens the possibility to use other laser wavelengths, for example, 364 nm, for holographic irradiation. The UV holographic recording and consequent post-processing result in the formation of smaller NPs. An average particle diameter was estimated as 3.2 nm.

Let us consider the mechanism of Ag NPs synthesis in details. Synthesis of Ag NPs in organic matrix, comprising silver salt and a reducing agent, consists of following stages: Ag^+^ reduction (Ag^+^ + e^−^ → Ag^0^ (atom)); Ag_*x*_ clusters formation (xAg^0^ → Ag_*x*_ (NP)); cluster aggregation and NP size growth [37,38]. It is known that the free radicals occurred when the initiator absorbs light, can serve as reducing agents for the metal ions. The mechanism of photo- and thermal reduction of Ag^+^ and the influence of various material components on the process requires an additional study. We can give only some considerations, which are consistent with the known data concerning the Ag^+^ reduction.

Absorbing the recording radiation, the CQ interacts with the MK that leads to the formation of aminoalkyl MK${}_{\left(-H\right)}^{•}$ and ketyl CQ${}_{\left(+H\right)}^{•}$ radicals:

(4) $$\begin{array}{c} \mathrm{MK}\;+\;\mathrm{CQ}\overset{h\nu }{\to }\mathrm{MK}{}_{\left(-H\right)}^{•}+\mathrm{CQ}{}_{\left(+H\right)}^{•} \end{array}$$

The aminoalkyl radicals MK${}_{\left(-H\right)}^{•}$ are active radicals that initiate a chain reaction of radical polymerization. In turn, the ketyl radicals CQ${}_{\left(+H\right)}^{•}$ can reduce Ag^+^ according to:

(5) $$\begin{array}{c} \mathrm{Ag}{}^{+}\;+\;H{}^{-}\;+\;O{}^{-}\overset{h\nu }{\to }\mathrm{Ag}{}^{0}\;+\;H{}^{+}\;+\;O{}^{-} \end{array}$$

The monitoring of the absorption spectra during thermal treatment shown that the heating was accompanied by a decrease in the intensity of the MK absorption band, i.e., by reducing of the MK concentration. This allows us to assume that MK${}_{\left(-H\right)}^{•}$ and CQ${}_{\left(+H\right)}^{•}$ radicals created through the thermally stimulated reactions play an important role in the thermally stimulated Ag reduction. Besides that the heating also stimulates the diffusion processes in the composite films that can also affect the activation of the reduction process.

### 3.3. Nonlinear Properties of Noble Metal NPs Gratings

Among the NPs of different natures, the NP of noble metals have the greatest influence on the nonlinear optical properties of the materials, which makes them potential candidates for design of high-speed nonlinear devices, for example, optical limiters and ultrafast optical switches [39,40,41].

Silver and gold NPs are most commonly used to create functional composite materials. Silver NPs are of particular interest because their absorption bands in the optical spectral region are characterized by a greater oscillator strength than that for Au NPs ([42] and references therein). Thus, higher nonlinear absorption coefficients can be expected for Ag NPs than for Au NPs under equal conditions. In addition the overlapping of plasmon and interband absorption bands is less pronounced for Ag NPs. For these NPs, the energy of the interband transition is approximately 4 eV (plasmon resonance energy is about 2.8 eV), while for Au NPs the energy of the interband transition is 2.3 eV. This makes it possible to investigate nonlinear optical effects caused solely by the contribution of surface plasmons at moderate excitation intensities.

There are a lot of articles related to the study of nonlinear properties of the nanocomposites based on Au and Ag NPs. The main results of studies of the nonlinear properties of nanocomposites containing Au NPs, which will be used in our work, are presented in ([5,43,44,45,46,47,48,49] and references therein). A fairly complete bibliography of works on the silver containing composites is given in ([50] and references therein).

#### 3.3.1. Nonlinear Characteristics of the Polymer Matrix with Au NPs

**Figure 10:**
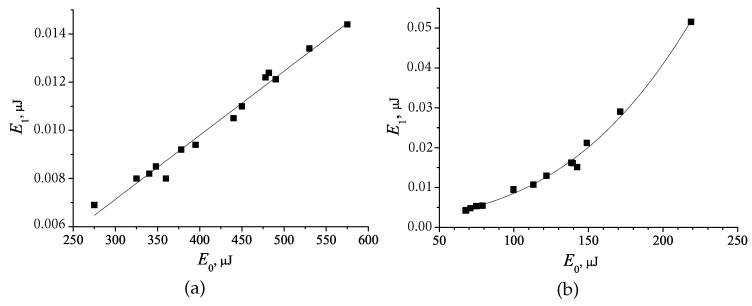
Nonlinear refractometry of Au NP nanocomposites containing Au1 (**a**) and Au2 (**b**) NPs.

Nonlinear characteristics of the nanocomposite with the Au NPs were measured by nonlinear refractometry [43]. The dependences of the energy of the laser pulse diffracted to the first order by the dynamic grating (E_1_) on the energy of the incident pulse (E_0_) are shown in Figure 10a,b.

Approximating the obtained data, it is possible to calculate the nonlinearity coefficient of the refractive index $n_{2}$ and, accordingly, the value of the third-order susceptibility $\chi^{\left(3\right)}$:

(6) $$\begin{array}{c} n_{2}\;=\;\frac{\lambda \;\eta^{1/2}}{\pi \;d\;I_{0}}\;\;\;\mathrm{and},\;\mathrm{correspondingly}\;\;\;\chi^{3}\left(-\omega ,\omega ;-\omega ,\omega \right)\;=\;\frac{4\varepsilon_{0}\;c\;n_{0}^{2}}{3}n_{2}, \end{array}$$

where λ is the excitation wavelength, η is the diffraction efficiency of dynamic gratings, *d* is the thickness of the nanocomposite layer, $I_{0}$ is the intensity of the beam, forming the grating, $\varepsilon_{0}$ is the dielectric constant of vacuum, *c* is the speed of light in vacuum, $n_{0}$ is the linear part of the nanocomposite refractive index.

Since the surface plasmon resonance band of the Au NP is located in the of 520–550 nm range, the excitation wavelength is placed within the plasmon absorption band. Despite the low concentration of Au NPs (1–2 wt. %) the formation of the dynamic gratings upon pulse exposure was clearly observed. For the Au1 NPs (size of 1.7 nm ± 0.3 nm) diffracted beam intensity depends linearly on the incident beam intensity (Figure 10a). For the composite with larger Au NPs (size of 2.7 ± 0.8 nm), the behaviour of this dependence changes. In this case there is a typical dependence $E_{1}$ = $AE_{0}^{3}$ for cubic nonlinear optical response, known for the Au NPs [43,44,45,46] (Figure 10b). This difference in the nonlinear optical response can be explained by the dependence of the electron energy quantization effect on the Au NP size. In [47] it was reported about decrease in cubic nonlinearity with a decrease in the size of Au NPs from 18 nm to 7 nm. In [48] the size of the Au NP of about 2 nm was defined as a critical dimension for the energy electron quantization. That is, at the size < 2 nm, the Au NPs are already considered as the nanoclusters, which possess significantly lower nonlinear optical parameters.

The calculated values are: $n_{2}$ ≈ 1.5 × 10^−7^ cm^2^/kW, $\chi^{\left(3\right)}∼$ 10^−8^ esu. The comparison of these results with the best values of cubic nonlinearity coefficients of metal NPs in various matrices, given in [5], indicates sufficiently large cubic optical nonlinearity of the structures under study. The comparable values of ($\chi^{\left(3\right)}∼$ 10^−7^ esu) was obtained by Ryasnyanskiy et al. [43] for the Au NPs in various matrices using magnetron sputtering method. Note that the concentration of Au NPs and their size in the studies mentioned above significantly exceed those used in our nanocomposite. Nevertheless, the nonlinear characteristics of the nanocomposite under study are slightly inferior to those achieved for various composites with Au NPs and can be improved by further optimization of the nanocomposite formulation.

**Figure 11:**
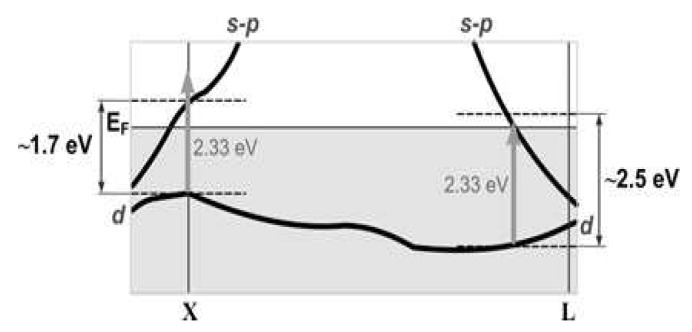
Energy level diagram explaining the mechanism of Au-nanocomposite optical nonlinearity.

A feature of the zone structure of Au NPs is that the energy of intraband excitation of the conduction electrons and the interband transition (2.3 eV) are close to the photon energy of the exciting radiation 2.33 eV (532 nm) (Figure 11). Therefore, as noted in [49], several mechanisms of cubic nonlinearity can be realized in Au NPs under different conditions. These mechanisms can be divided into fast electron or Kerr’s and relatively slow ones. The latter are associated with the heat transfer from the heated Au NPs to the environment. The intraband transitions of conduction electrons may contribute to the nonlinear response. According to [49] for spherical Au NPs the value of $\chi^{\left(3\right)}$ can reach ∼ 10^−13^ esu, which is significantly lower than the values obtained by us. Another mechanism is associated with the interband electron transitions between *d* and s−p zones. This contribution has a negative sign [49]. The third fast mechanism is the contribution of hot electrons to the nonlinear response. If the wavelength of the laser radiation is close to the maximum of the plasmon resonance, as in our case, this mechanism can give a significant positive, unlike the previous two mechanisms, contribution to the nonlinear absorption and affect the efficiency of the dynamic grating.

When the sample is exposed to laser radiation, the Au NPs are also heated with the following heat-transfer to the surrounding medium. This leads to a nonlinear change in the *n* of the sample. This is a comparatively slower effect. The estimation of the relaxation time for some composite materials doped with the Au NPs in [43] gave the value of the thermal relaxation time of $\tau_{r}$≃ 6 ns. Since the pulse duration of the laser used is 10 ns, which is comparable to the typical thermal relaxation time, the thermal mechanism can not be discarded. At the same time, a cumulative heat-effect can be ignored, because of the low repetition rate of laser pulses 0.5 Hz.

#### 3.3.2. Nonlinear Characteristics of the Polymer Matrix with Ag NPs

The Z-scan method was used to study the nonlinear optical properties of the polymer matrix with Ag NPs. The real and imaginary parts of the complex susceptibility of the third order $\chi^{\left(3\right)}$, the nonlinear absorption coefficient β and the coefficient of refractive index nonlinearity $n_{2}$ were determined by the method proposed by Sheik-Bahae et al. [51].

**Figure 12:**
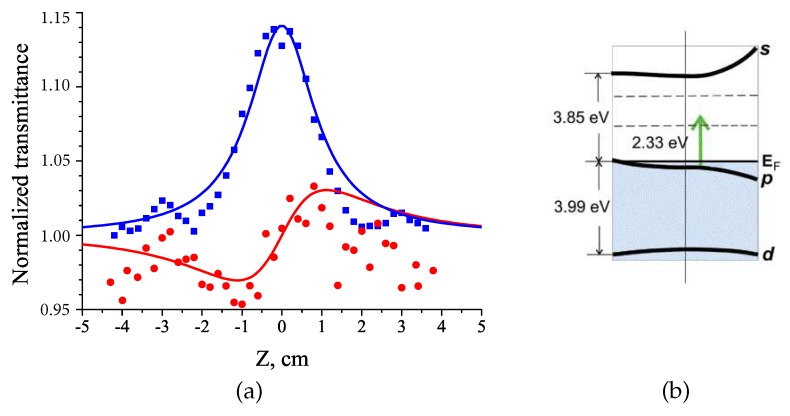
Normalized open-aperture (blue squares) and limited-aperture (red circles) Z-scan traces of Ag-nanocomposite at 532 nm excitation ($\tau_{p}$ = 20 ns, f = 0.5 Hz, $I_{0}$ = 5.11 MW/cm^2^). The lines are the theoretical fits (**a**). Energy level diagram explaining the mechanism of Ag-nanocomposite optical nonlinearity (**b**).

Figure 12a shows the transmission curves obtained by Z-scan with an open and limited aperture when the structure is excited by nanosecond radiation with an intensity of $I_{0}$ = 5.1 × 10^6^ W/cm^2^. The normalized transmittance increases at the focal point (z = 0 mm), indicating the saturation of optical absorption. The decrease in the absorption under strong optical irradiation corresponds to a negative value of Im $\chi^{\left(3\right)}$ and β at the specified $I_{0}$ irradiation.

The normalized transmittance obtained with the limited aperture Z-scan scheme shows a pure nonlinear refractive effect. The character of the curves indicates the self-focusing of laser radiation in the layer of Ag-doped polymer nanocomposite, i.e., the positive value of the nonlinear refractive index. The asymmetry of the transmission curves obtained with a limited aperture may be the result of a large nonlinear phase shift caused by the action of pulsed laser [52]. This phenomenon requires an additional analysis and verification.

The measurements were repeated several times to ensure the results reproducibility. In our experiments no irreversible changes in transmission were observed. The measurements under the same conditions with the pure polymer matrix have shown no nonlinear response.

The calculated values of Im $\chi^{\left(3\right)}$, Re $\chi^{\left(3\right)}$, β and $n_{2}$ are collected in Table 2.

The obtained values of the nonlinear parameters of the proposed nanocomposite coincide in order of magnitude and, in some cases, by one order of magnitude higher than the values reported by other authors using the NPs of a similar size and under similar excitation conditions (works cited in [50]).

We can explain the mechanism of optical nonlinearity in terms of electronic transitions in Ag NPs. The scheme of the energy levels proposed by Rosi et al. [53] is shown in Figure 12b. Noble metals are characterized by a valence band, formed by fully populated *d*-states, and a conduction band, formed by s−p states and filled up to the Fermi level. For the Ag NPs, the energy of the interband transition is equal approximately to 3.99 eV (310 nm). The distance p→s between the populated *p*-states and the free *s*-states of the conduction band is of about 3.85 eV (322 nm). The plasmonic absorption band of metallic NPs is associated with the collective oscillations of free electrons, which occupy energy states near the Fermi level in the conduction band. The plasmonic resonance for our nanocomposite is of about 2.87 eV. In our experiments the energy of the photons, used for excitation of the nanocomposites, is equal to 2.33 eV (532 nm) that was significantly less than the energies of the interband and p→s transitions. Therefore, one-photon excitation of hot electrons at the wavelength of 532 nm causes the saturated absorption. Thus, we can conclude that under the used experimental conditions the nonlinear response of the studied structures is the result of intraband transition with hot electron excitation.

## 4. Conclusions

In this paper we report our recent investigations of new photopolymerizable nanocomposite materials based on acrylate polymers and noble metal nanoparticles. These nanocomposites are suitable to create effective periodic 1D, 2D arrangement of Au and Ag NPs in polymer matrices. They show excellent holographic characteristics as well as nonlinear optical properties. The material composition was chosen by applying the known model of a two-component photocurable mixture, consisting of multifunctional and monofunctional acrylic monomers, which provides a spatial diffusion NP redistribution in low-viscous matter upon holographic exposure. A monofunctional monomer can be replaced by a chemically neutral component. We have shown that this approach can be used to create holographic periodic structures formed by the ready NPs introduced into the monomer mixture (the *ex situ* method) or by the NPs synthesized directly in a polymer matrix from a pre-ordered metal precursor presented in the mixture (the *in situ* method).

We have improved the composite including the Au NPs which, despite the small NP concentration, provides the refractive index modulation, Δn = 2 $n_{1}$, of about 0.015 in the case of the NPs of a 1.7 ± 0.36 nm size and Δn of approximately 0.017 in the case of the Au NPs with larger size, 2.7 ± 0.94 nm.

We have developed a new composite for the fabrication of ordered submicrometer-scale structures containing Ag NPs with the in situ method. These structures possess high, up to 0.042, value of Δn. To our knowledge, this value is the highest reported in academic literature not only for the nanocomposites containing metal NPs, but also for the NPs of another nature (metal oxides, semiconductors, etc.).

Using theoretic model of the periodic structures formation, the experimental data on the kinetics of the grating holographic recording, as well as the method of IR spectroscopy, we have found that the Au NPs play a double-functional role in the grating formation. The NPs not only participate in diffusion, but also significantly affect the polymerization process, increasing the polymer conversion depth. This leads to the additional displacement of a monofunctional monomer from the polymer network and, accordingly, to the growth of the Δn. Increasing the particle size leads to the amplification of these processes. The appearance of the peaks corresponding to the excitation of localized surface plasmons in Au NPs in the absorption spectrum of the composite containing the Au NPs with a size of 2.7 ± 0.94 nm suggests that the local field amplification may also affect the polymerization process and increase Δn.

The in situ method to obtain a periodic distribution of silver NP in polymer matrix combines the holographic ordering of a metal precursor in polymer matrix and the photo-/thermo-induced reduction of metal NPs in the areas predominantly enriched with an Ag-precursor. The polymerization induced phase separation of the composite components and a diffusion Ag precursor transfer to the unlit regions of the film is the basis of the periodic structures formation mechanism. The use of the new photocurable monomer blend in the composite enhances the segregation of the precursor solution from the polymer matrix that increases $n_{1}$ by 1.5 times. The influence of the forming polymer matrix on the size and localization of NPs was established. Even though the depth of the precursor modulation is less than 100%, the Ag NPs are localized mainly in the unlit areas of the layer and practically absent in the lit areas. The size of the NPs can be controlled with the utilized photoinitiator and the wavelength of the holographic recording. Replacement of a photoinitiator in the composite formulation and application of UV holographic exposure results in smaller, of about 3.2 nm, Ag NPs. Thus, this makes it possible to control the parameters of periodic structures and, accordingly, their plasmonic and nonlinear optical properties.

Both types of the nanocomposites possess nonlinear optical properties. In the case of the Au NPs doped nanocomposites the nonlinear response occurs only by using the NPs with a size of 2.7 nm, in which the plasmon resonance is observed. When the NPs size is reduced to 1.7 nm, the response becomes linear and surface plasmon resonance band in such the nanocomposites is not detected. This is in line with the known statement that the phenomenon of dimensional quantization does not appear of the metallic gold clusters with a size ≤2 nm.

The nonlinear response of the nanocomposite with Ag NPs is determined by the absorption saturation. The nonlinear refractive index has a positive sign, which leads to self-focusing of laser radiation in the nanocomposite layer. The values of the nonlinear parameters calculated using the experimental measurements in some cases are one order of magnitude higher than those obtained by other authors for NPs of similar size and under similar excitation conditions. Generally, the nonlinear response of the developed nanocomposites with Ag and Au NPs are explained by the local field amplification due to excitation of hot electrons in the conduction band.

The obtained results indicate that the presented metal-polymer nanocomposites are prospective materials for optoelectronics, optical sensing, biomedicine, etc.

## Figures and Tables

**Table 1:** Comparison of theoretical and experimental results for the model in Figure 5.

Model			Experiment	
Description	*n*_1_	$\Delta \upsilon_{P1}^{*}/\upsilon_{P1}$	*n*_1_	$\Delta \upsilon_{P1}^{*}/\upsilon_{P1}$
Polymer P1 (EBPDA) is fully localized in the	0.0157	100%	0.0027	17%
illuminated areas (composite without NPs)				
Polymer P1 (EBPDA) is fully localized in the				
illuminated areas	0.0167	100%		
NPs are fully localized in the dark areas			0.0073 Au1	38%
The modulation of P1 (EBPDA) concentration			0.0084 Au2	42%
is the same as in a composite without NPs	0.0037	17%
NPs are fully localized in the dark areas				
The modulation of P1 (EBPDA) concentration				
is absent	0.001	0%		
NPs are fully localized in the dark areas				

**Table 2:** Nonlinear optical characteristics of polymer layer with Ag NPs.

$n_{2}$, cm^2^/W	β, cm/W	Re$\chi^{\left(3\right)}$, esu	Im$\chi^{\left(3\right)}$, esu	$\left|\chi^{\left(3\right)}\right|$, esu
$3\times 10^{-10}$	$-1.13\times 10^{-4}$	$1.36\times 10^{-8}$	$-2.88\times 10^{-8}$	$3.19\times 10^{-8}$
